# Comparison of different positioning techniques for reduction of induced vertical deviation following Nishida procedure for the treatment of sixth nerve palsy

**DOI:** 10.1371/journal.pone.0329139

**Published:** 2025-07-30

**Authors:** Chong-Bin Tsai, Chien-Liang Fang

**Affiliations:** 1 Department of Ophthalmology, Ditmanson Medical Foundation Chia-Yi Christian Hospital, Chia-Yi City, Taiwan; 2 Department of Optometry, College of Medical and Health Science, Asia University, Taichung City, Taiwan; 3 Division of Plastic and Reconstruction Surgery, Department of Surgery, Ditmanson Medical Foundation Chia-Yi Christian Hospital, Chia-Yi City, Taiwan; 4 Department of Food Nutrition and Health Biotechnology, College of Medical and Health Science, Asia University, Taichung City, Taiwan; Aravind Eye Hospital, INDIA

## Abstract

**Purpose:**

Induced vertical deviation is a potential complication following the Nishida procedure for the treatment of sixth nerve palsy. This study aims to compare different positioning techniques for the reduction of this complication.

**Methods:**

We retrospectively examined medical records from consecutive patients who underwent the Nishida procedure, classifying them into three positioning groups: intra-quadrant (IQP), lateral rectus border (LRBP), and horizontal meridian (HMP). Surgical and pre/postoperative data were compared.

**Results:**

Among the 27 included patients (8 IQP, 9 LRBP, 10 HMP), all three groups demonstrated similar reductions in esodeviation: IQP, 44.0 ± 18.7 Prism Diopters (PD); LRBP, 42.2 ± 15.3 PD; HMP, 42.2 ± 7.8 PD; (P = 0.675). After surgery, one patient in the IQP group developed hypertropia of 18 PD, necessitating a secondary surgery to treat the vertical diplopia. In the LRBP group, two patients had hypotropia of 30 PD and 10 PD, respectively, and one patient had hypertropia of 6 PD. In the HMP group, one patient initially had hypertropia of 2 PD, which resolved during subsequent follow-up. A lower incidence of induced vertical deviation was observed in the HMP (10%) and IQP (13%) groups compared to the LRBP group (33%). However, this difference did not reach statistical significance due to the small sample size.

**Conclusion:**

There is no statistically significant difference among the three positioning techniques (IQP, LRBP, HMP) in the correction of esodeviation and reduction of incidence of induced vertical deviation following Nishida procedure.

## Introduction

After Hummelsheim’s [1] pioneering work in 1907, several different vertical rectus muscle transposition (VRT) techniques have been developed to treat esodeviation resulting from sixth nerve palsy. These methods include partial tendon rectus muscle transposition [1], full tendon rectus muscle transposition [2], rectus muscle union [3] single rectus muscle transposition [4,5], and Nishida procedure [6,7]. Induced vertical deviation is one of the potential complications following VRT surgery and can lead to troublesome vertical diplopia, compensatory head positioning, and, in persistent cases, may require additional surgical interventions. It has been observed in 0% − 40% after VRT procedures [8]. Several strategies have been suggested to reduce the occurrence of induced vertical deviation in VRT surgery. These approaches include self-adjusting vertical muscle union either underneath or over the lateral rectus muscle [9,10], adjustable vertical rectus muscle transposition [11], single posterior fixation suture [12], crossed-adjustable transposition [13], and intraoperative monitoring of torsion [14]. These methods are mainly applicable to full or partial tendon transpositions, yet they are not focused on Nishida procedure.

Nishida and colleagues’ introducing the novel muscle transposition procedure without tenotomy in 2003 [6]. They subsequently modified the procedure to only suture the temporal margins of the muscles to the sclera, without performing tenotomy or muscle splitting (the Nishida procedure) [7]. In the procedure, the suture sites were placed at the midpoints of the superotemporal and inferotemporal quadrants. It is worth noting that, during surgery, the locations of these quadrants’ midpoints on the globe are often selected arbitrarily by the surgeon. This arbitrary positioning may result in unbalanced vertical forces, potentially leading to induced vertical deviation.

In this study, we compare three different positioning techniques for the Nishida procedure and assess their effect on correction of esodeviation and reduction of induced vertical deviation.

## Materials and methods

This study adhered to the principles of the Declaration of Helsinki and was approved with waiver of informed consent by the Institutional Review Board of the Ditmanson Medical Foundation Chiayi Christian Hospital (approval ID number IRB2023115). We conduct a retrospective review of medical records for consecutive patients who underwent the Nishida procedure at the Department of Ophthalmology of Chiayi Christian Hospital between January 2020 and December 2023. The inclusion criteria of the study were (a) patients who had been diagnosed with unilateral sixth nerve palsy and had not experienced recovery after a minimum follow-up period of 6 months; (b) patients who underwent unilateral Nishida procedure as the primary surgery. The exclusion criteria were (a) previous strabismus surgery of the affected eye; (b) additional botulinum toxin injection to the medial rectus muscle during surgery; (c) patients with preoperative vertical deviation.

The data was accessed for research purposes on 20/01/2024. The following patient data was extracted from the records: sex, age at surgery, laterality, etiology of the sixth nerve palsy, the duration between onset of diplopia and surgery, prior strabismus surgery, pre- and postsurgical primary angle of deviation for near and distance, preoperative abduction deficit and follow-up time. Operation notes were reviewed for the restriction of abduction detected by the intraoperative forced duction test, the type of positioning technique used during surgery, and the type and amount of procedure on each muscle.

**Fig 1:**
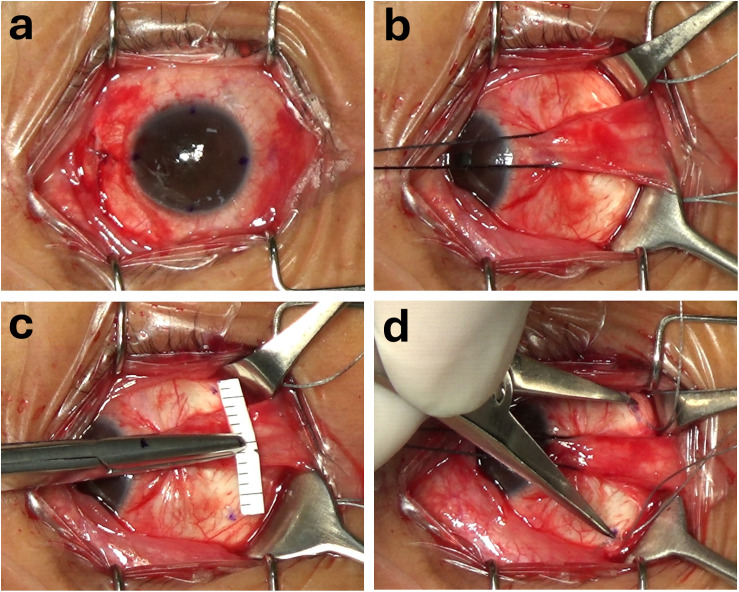
The horizontal meridian positioning technique. **(a)** Limbal markings were made at 3, 6, 9 and 12 o’clock as references for the horizontal and vertical meridians of the globe. **(b)** Two fornix incisions were performed in the superotemporal and inferotemporal quadrants to approach the superior and inferior rectus muscles, respectively. **(c)** A straight Mosquito hemostatic forceps securing a pre-cut paper ruler was used as a T-shaped measurement tool. The straight forceps was aligned with the 3 and 9 o’clock limbal marks to establish the horizontal meridians. The desired distance was marked on the surface of one of the jaws of the straight forceps and the paper ruler was used to measure the perpendicular distance from the horizontal meridian. **(d)** Following marking, the precise distance between the two marks was double-checked with a caliber.

All patients underwent the Nishida procedure as described by Nishida and colleagues [7], and all surgeries were performed by a single experienced surgeon (CBT). Under general anesthesia, conjunctival and limbal markings and measurements were performed intraoperatively with the patient in a supine position. Intraoperative forced duction test was performed to evaluate the restriction of abduction due to contracture of the medial rectus muscle. Limbal markings were made at 3, 6, 9 and 12 o’clock as references for the horizontal and vertical meridians of the globe. (Fig 1a) A fornix incision in the inferonasal quadrant was performed. The medial rectus muscle was recessed with a sliding noose adjustable technique using a double-armed 6−0 polyglactin 910 suture (Vicryl, Ethicon, Somerville, NJ). The amount of recession was decided according to the contracture status of the medical rectus muscle. Another two fornix incisions were performed in the superotemporal and inferotemporal quadrants to approach the superior and inferior rectus muscles, respectively. (Fig 1b) For the transposition, a 5−0 polyester braided fiber suture (Ethicon, Somerville, NJ) was passed through the temporal one-third of each vertical rectus muscle at a distance of predetermined amount posterior to its insertion. Subsequently, a positioning technique was employed to locate the site for suture placement (the anchoring site) to the sclera and the suture was tightened to transpose the vertical muscles.

**Fig 2:**
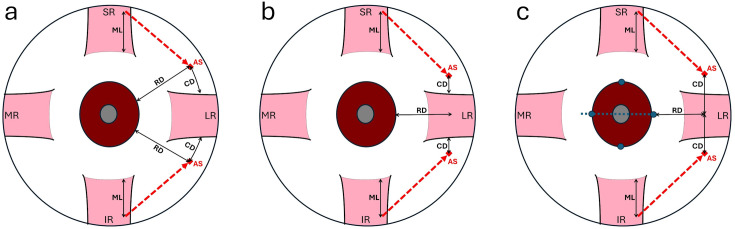
Schematic drawing of three positioning techniques. A suture was passed through muscle at a distance (ML) from insertion to transpose (red dotted line) the muscle and was anchored at the anchoring site (AS), which was located with three different positioning techniques: **(a)** Intra-quadrant positioning (IQP), **(b)** Lateral rectus border positioning (LRBP) **(c)** Horizontal meridian positioning (HMP). CD, circumferential displacement of the anchoring site; ML, length of the muscle to be transposed; RD, radial distance from limbus to the anchoring site; SR, superior rectus, LR, lateral rectus; IR, inferior rectus; MR, medial rectus.

There were three parameters of the transposing process, including the distance (ML) measured from the insertion to where the suture was passed through the vertical rectus muscle to be transposed; the radial distance (RD) from limbus to the anchoring site; and circumferential displacement (CD), depending on different positioning techniques, which could be measured from the muscle border or from the horizontal meridian to the anchoring site. (Fig 2) Regarding the technique of positioning the anchoring site, the patients could be categorized into the following three groups.

### Intra-quadrants positioning (IQP)

In the intra-quadrant positioning (IQP) group, the anchoring site for the suture was placed at a radial distance (RD) from the limbus. Circumferentially, it was positioned midway between the vertical rectus muscle and the lateral rectus muscle (CD = 1/2). In some patients, to augment the transposition effect, the anchoring site was placed closer to the lateral rectus: either at one-quarter of the distance (CD = 1/4), or directly adjacent to it (CD = 0). (Fig 2a) To ensure symmetric placement and avoid globe rotation during marking, both the superotemporal and inferotemporal quadrants were exposed simultaneously using two retractors,

### Lateral rectus border positioning (LRBP)

In the lateral rectus border positioning (LRBP) group, the anchoring site for the suture was placed at a radial distance (RD) from the limbus and at a distance (CD) from the borders of the hooked lateral rectus muscle circumferentially. (Fig 2b) When marking the anchoring site, both corners of the insertion of the lateral rectus muscle were grasped with two locking forceps simultaneously to avoid asymmetric hooking of the lateral rectus muscle with using a single muscle hook.

### Horizontal meridian positioning (HMP)

**Fig 3:**
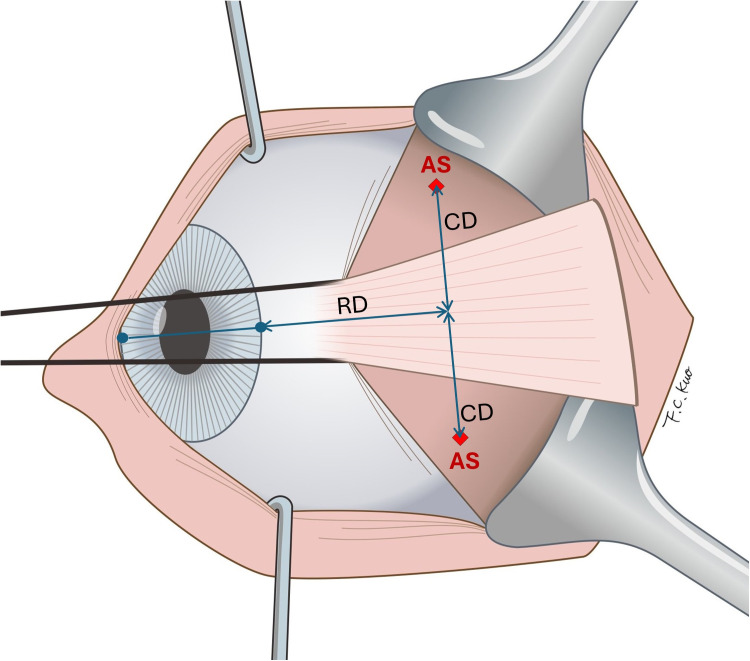
Schematic drawing of the alignment of horizontal meridian and positioning the anchoring site during surgery. AS, anchoring site; CD, circumferential displacement of the anchoring site; RD, radial distance from limbus to the anchoring site.

In the horizontal meridian positioning (HMP) group, the anchoring site of the suture was placed at a radial distance (RD) from the limbus and at a distance (CD) from the horizontal meridian circumferentially. (Fig 2c and 3) In practice, because the conjunctiva covering the lateral rectus muscle are freely movable and cannot be used as a reliable marking surface, we developed a horizontal meridian positioning technique for marking on the sclera. This technique involved using a straight Mosquito hemostatic forceps to secure a pre-cut paper ruler, creating a simple T-shaped measurement tool. The straight forceps was aligned with the 3 and 9 o’clock limbal marks to establish the horizontal meridians. The desired distance of RD was marked on the surface of one of the jaws of the straight forceps and the paper ruler was employed to measure the perpendicular distance of CD from the horizontal meridian. (Fig 1c) Following marking, the precise distance between the two marks was double-checked using a caliber. (Fig 1d)

Ocular alignment was assessed at 1–3 hours after surgery using alternative cover test or Krimsky test. Adjustments were made to the medial rectus muscle when needed. The postoperative deviation at the primary position was reviewed at 1 month after surgery and at the last follow-up. The amount of correction was calculated from the difference between preoperative and postoperative deviations.

### Statistical analysis

All the statistical analyses were conducted using IBM SPSS Statistics for Windows (Version 28.0, IBM Corp, Armonk, NY). Continuous variables are presented as mean and standard deviation with range, while categorical variables are presented as numbers and percentages. Kruskal-Wallis test was applied to compare the continuous variables among groups. Pearson correlation was used to examine the correlation between continuous variables. Categorical variables among groups were compared via Pearson Chi-square test. A P value of 0.05 was chosen as the level of statistical significance.

## Results

A total of 35 patients who were diagnosed as sixth nerve palsy and underwent the Nishida procedure during the study period were selected initially. However, 5 were excluded due to previous surgery and 3 to bilateral abducens palsy. The clinical characteristics of the 27 patients in the final study population are summarized in Table 1. Original data are shown in S1 Table. There were 14 (52%) male and 13 (48%) female patients. The mean age at surgery was 31.3 ± 23.2 years (range 3–71). The presented etiologies included idiopathic in 16 (59%), tumor in 6 (22%), congenital in 3 (11%), hemorrhagic in 1 (4%), and trauma in 1 (4%). The duration between onset of diplopia and surgery was 7.8 ± 13.8 years (range 1–68). Of these patients, 8 were categorized into the IQP group, 9 into the LRBP group, and 10 into the HMP group. The characteristics of three groups were comparable except for the eye affected.

**Table 1:** Patient characteristics (N = 27).

	Group			P value
	IQP (n = 8)	LRBP (n = 9)	HMP (n = 10)	
Age at surgery, mean ± SD (range), year	28.8 ± 25.4 (4-69)	36.9 ± 21.9 (3-66)	28.2 ± 24.0 (5-71)	0.712
Sex, Female Male	5 (62.5%) 3 (37.5%)	5 (55.6%) 4 (44.4%)	3 (30.0%) 7 (70.0%)	0.337
Eyes affected, Right Left	2 (25.0%) 6 (75.0%)	3 (33.3%) 6 (66.7%)	8 (80.0%) 2 (20.0%)	0.037
Etiology, no (%)				
Idiopathic	3 (38%)	6 (67%)	7 (70%)	0.111
Tumor	1 (12%)	2 (22%)	3 (30%)	
Trauma	1 (12%)	0	0	
Congenital	3 (38%)	0	0	
Hemorrhage	0	1 (11%)	0	
Time from onset to surgery, mean ± SD (range), year	13.4 ± 22.9 (1-68)	4.9 ± 8.0 (1-26)	5.9 ± 6.8 (1-20)	0.376
Follow-up, mean ± SD (range), month	8.6 ± 10.5 (1-33)	8.3 ± 5.5 (2-18)	3.6 ± 3.9 (1-13)	0.079

HMP, horizontal meridian positioning; IQP, intra-quadrant positioning; LRBP, lateral rectus border positioning, SD, standard deviation

The surgical data and outcomes are summarized in Table 2. The mean preoperative esodeviation, which measured 46.0 ± 13.8 Prism Diopters (PD) (with a range of 30–85), showed no statistically significant differences among the three groups. Both the preoperative abduction deficit and the intraoperative abduction deficit assessed through the forced duction test exhibited comparable results across all groups. The IQP group exhibited a notably reduced medial rectus recession compared to other groups. One potential explanation is that the IQP technique was used during the early phase of the study period when the surgeon adopted a more conservative approach about the surgical dosage.

**Table 2:** Surgical data and outcomes (N = 27).

	Group			P value
	IQP (n = 8)	LRBP (n = 9)	HMP (n = 10)	
Preoperative esodeviation^a^ at distance, mean ± SD (range), PD	44.1 ± 9.7 (30-60)	47.2 ± 19.2 (30-85)	46.5 ± 12.0 (35-75)	0.875
Preoperative vertical deviation, mean ± SD (range), PD	0 ± 0 (0)	0 ± 0 (0)	0 ± 0 (0)	
Preoperative abduction deficit, mean ± SD (range), grading	−2.1 ± 1.5 (−5 - −1)	−1.8 ± 2.3 (−7–0)	−2.3 ± 2.0 (−6 - −1)	0.583
Intraoperative abduction deficit by forced duction test, mean ± SD (range), grading	−1.2 ± 0.8 (−2–0)	−2.0 ± −1.2 (−4–0)	−1.4 ± 1.7 (−5–0)	0.396
Medial rectus recession, mean ± SD (range), mm	3.0 ± 2.0 (0-5)	5.2 ± 1.3 (3-7)	4.9 ± 1.2 (4-7)	0.035
ML, mean ± SD (range), mm	9.0 ± 1.7 (8-10)	8.6 ± 0.9 (8-10)	8.9 ± 0.9 (8-10)	0.572
RD (No.)	10 mm (7) 12 mm (1)	10 mm (9)	10 mm (10)	
CD (No.)	1/2 (2) 1/4 (3) 0 (3)	3 mm (7) 2 mm (1) 0 mm (1)	7 mm (9) 8 mm (1)	
Postoperative esodeviation at distance, mean ± SD (range), PD	0.1 ± 11.5 (−25–16)	5.0 ± 10.1 (−2-25)	4.3 ± 9.1 (−2–25)	0.952
Reduction of esodeviation, mean ± SD, PD	44.0 ± 18.7 (24-85)	42.2 ± 15.3 (30-70)	42.2 ± 7.8 (34–52)	0.675
Postoperative vertical deviation at 1 month, mean ± SD (range), PD	2.3 ± 6.4 (0-18)	5.1 ± 10.0 (0-30)	0.2 ± 0.6 (0-2)	0.342
No. of induced vertical deviation at 1 month	1 (13%)	3 (33%)	1 (10%)	0.371
Postoperative vertical deviation at final follow-up, mean ± SD (range), PD	1.5 ± 4.2 (0-12)	3.6 ± 5.9 (0-16)	0 ± 0 (0)	0.141
No. of induced vertical deviation at final follow-up	1 (13%)	3 (33%)	0 (0%)	0.303

CD, circumferential displacement of the anchoring site; HMP, horizontal meridian positioning; IQP, intra-quadrant positioning; LRBP, lateral rectus border positioning; ML, length of the muscle to be transposed; PD, prism diopters; RD, radial distance from limbus to the anchoring site; SD, standard deviation.

^a^ A plus sign (+) was assigned to an esotropic angle, and a negative sign (-) was assigned to an exotropic angle.

Regarding the positioning technique, the length of the rectus muscle (ML) and the radial distance (RD) of the anchor point from limbus was similar among groups. The choice of circumferential displacement (CD) for the anchor point differed in each group. In IQP group, half distance (midpoint) was used in 2 patients, quarter distance in 3 patients, and near approximate the LR in 3 patients. In LRBP group, 3 mm displacement from the LR muscle border was chosen in 7 patients, 2 mm in 1 patient, and 0 mm in 1 patient. In HMP group, 7 mm displacement from horizontal meridian was used in 9 patients and 8 mm in 1 patient.

After surgery, all three groups exhibited comparable reduction of esodeviation (IQP, 44.0 ± 18.7 PD; LRBP, 42.2 ± 15.3; HMP, 42.2 ± 7.8; P = 0.675). Concerning the induced vertical deviation, 1 patient in IQP group had hypertropia of 18 PD. Although the hypertropia was reduced to 12 PD after 5 months. The patient had to receive a recession of superior rectus muscle to eliminate the bothersome vertical diplopia. In LRBP group, there were hypotropia of 30 PD and 10 PD in 2 patients separately and hypertropia of 6 PD in 1 patient. The hypotropia of 30 PD was later reduced to 16 PD after 14 months. All three patients still experienced vertical diplopia at their final follow-up. In the HMP group, 1 patient had hypertropia of 2 PD, which subsided in subsequent follow-up. However, due to the small number of patients in this study, the difference could not reach a level of statistical significance.

## Discussion

We evaluated three positioning techniques for the Nishida procedure, focusing on their effectiveness in correcting esodeviation and reducing induced vertical deviation. Among 27 patients, the average correction was 42.7 ± 13.7 PD, closely matching Nishida’s original report of 42.4 ± 10.9 PD [6]. All our cases included medial rectus recession, compared to only 4 of 10 in Nishida’s series. A 2013 study by Muraki et al. [15] reported a slightly higher correction (46.3 ± 13.1 PD) with medial rectus recession in 6 of 9 cases. Later studies showed corrections ranging from 46.9 to 69.8 PD [16–20], likely due to larger medial rectus recessions of 6.1 mm in Hernandez-Garcia et al. [19] and 7.8 mm in Yao et al. [20], compared to 4.4 ± 1.7 mm in our series.

In our study, induced vertical deviation occurred more frequently in the LRBP group (33%) than in the HMP (10%) and IQP (13%) groups, though the difference was not statistically significant. Previously published studies on the Nishida procedure can be categorized into two types of positioning techniques. The first, the IQP type, places anchoring sites within each quadrant of the globe. Nishida’s 2003 study [6] and later modifications [15], as well as Sabermoghadam et al.’s [16] approach using more posterior placement (16 mm from the limbus), all reported no vertical deviation. The second, the LRBP type, positions sites near the lateral rectus muscle border. Yao et al. [17,18,20] reported vertical deviations of 14%, 8%, and 25% across three studies using this method. These findings suggest a higher risk of induced vertical deviation with the LRBP technique, consistent with our results.

Both previously mentioned positioning techniques involve using visible landmarks on the globe. In the LRBP technique, the surgeon utilizes the borders of the lateral rectus muscle’s belly. However, the borders can be subject to displacement and unreliability due to factors such as tilt hooking, globe rotation, and passible pathological muscular changes. Particularly in sever sixth nerve palsy cases, the lateral rectus muscle often becomes thin and atrophic, and may add potential bias when be used as landmarks.

In the IQP technique, surgeons aim to locate the midpoint of each globe quadrant, but in practice, this often means the midpoint of the distance between adjacent rectus muscle insertions. These distances, however, are not uniform. Apt [21] found the distance from the superior to lateral rectus averages 7.1 ± 0.8 mm, while lateral to inferior rectus is 8.0 ± 0.8 mm. As a result, the “midpoints” are asymmetrically placed, especially when anchoring 10–12 mm from the limbus. Though rectus insertions are more reliable than muscle borders, this asymmetry introduces potential bias.

Despite anatomical variation, rectus muscle insertions are generally more consistent than rectus muscle borders, likely explaining the lower incidence of vertical deviation with the IQP technique compared to the LRBP. However, the lateral rectus muscle is wider at its insertion, and part of it may be obscured by the muscle hook during surgery. Surgeons should be cautious using the insertion corners as landmarks, which may contribute to the “learning curve” in the vertical rectus transposition surgery noted by Rosenbaum [22].

To minimize bias from anatomical structures variability, we developed the HMP technique using an imaginary horizontal meridian to identify symmetrical anchoring points in both quadrants of the globe. However, marking directly on the movable conjunctiva proved impractical. Current corneal markers were also unsuitable for making 10 mm scleral markings from the limbus. To overcome this, we created a simple T-shaped tool using common operating room materials. While effective, it requires skill to mark accurately on the curved eye surface. A custom-designed device may offer a better solution in the future.

One potential drawback in our technique is the marking of the horizontal meridian while the patient is in a supine position and under general anesthesia. Research indicates that transitioning from a seated to a supine position can result in an average cyclotorsion of around 3°, with a range spanning from −7° to 14° [23]. The impact of general anesthesia on cyclotorsion remains uncertain. Consequently, there is a possibility that the horizontal meridian may not remain truly “horizontal” once the patient recovers from anesthesia and returns to a seated position. Further research is needed to determine the extent to which this factor may influence the induced vertical deviation.

The HMP technique aims to improve symmetry when identifying the anchoring site for the Nishida procedure. However, the link between symmetry and induced vertical deviation remains debated. Holmes et al. [14] suggested that anatomical differences between the superior and inferior rectus muscles, along with mechanical and connective tissue factors, may affect transposed muscle force. They proposed that even precise symmetry could cause unintended vertical deviation, especially if the inferior rectus is relatively tight. In fact, they argued that perfect symmetry might not be ideal in vertical rectus transposition (VRT) surgery. Consequently, in augmented VRT, the posterior fixation suture is placed 2 mm from the inferior border of the lateral rectus for the inferior rectus, while it is aligned with the superior border for the superior rectus. In contrast, del Pilar Gonzalez & Kraft [24] recommended inserting the superior rectus 2 mm above the superior border to reduce postoperative hypertropia in full VRT.

Connective tissue also plays a role. Rosenbaum [22] warned that excessive lateral slippage of the vertical rectus muscle can cause vertical deviation. To prevent this, he advised limiting septum dissection to 10 mm behind the muscle insertion and verifying the final lateral position. Ruth et al. [25] attributed induced hypotropia to incomplete severance of attachments between the inferior rectus and surrounding tissues, particularly the capsulopalpebral fascia and Lockwood’s ligament. They emphasized removing all nasal capsulopalpebral attachments to avoid lateral restriction during transposition.

## Conclusion

This study compared three anchoring site positioning techniques used in the Nishida procedure for sixth nerve palsy. While all three methods achieved similar effectiveness in correcting esodeviation, the HMP technique demonstrated the lowest incidence of induced vertical deviation, followed by IQP and LRBP. Although the differences were not statistically significant, the trend suggests that HMP may better minimize vertical misalignment postoperatively. There are certain limitations in our study, including its retrospective nature and a relatively small sample size. The secondary angle of deviation was not measured. Surgical procedures in the three groups were performed at different time intervals without randomization for technique selection, potentially introducing variations in surgical experience and bias. Additionally, the follow-up duration, particularly for the HMP group, was relatively short. Despite these limitations, our findings suggest promise for the HMP technique in reducing undesired vertical deviations during the Nishida procedure for sixth nerve palsy, warranting further investigation.

## Supporting information

S1 Table Original data. (DOCX)
