# Supplementary material for: Comparison of different positioning techniques for reduction of induced vertical deviation following Nishida procedure for the treatment of sixth nerve palsy
Source: PLoS One. 2025 Jul 30;20(7):e0329139. doi: 10.1371/journal.pone.0329139 (PMC12309997; doi:10.1371/journal.pone.0329139)
Supplement: S1 Table — (DOCX) [file pone.0329139.s001.docx]

**S1 Table. Original data.**

| No | Group | Etiology | R/L | Onset to op  (y) | Preop  ET angle  (PD) | Preop ABD Deficit | FDT | MRc  (mm) | ML  (mm) | RD  (mm) | CD | Postop  1 month  Deviation | Postop  1 month  Vertical Diplopia | Final F/U  Deviation | Final F/U Vertical Diplopia | F/U (Months) |
| --- | --- | --- | --- | --- | --- | --- | --- | --- | --- | --- | --- | --- | --- | --- | --- | --- |
| 1 | IQP | Trauma | R | 5 | 48 | -5 | NA | 4 | 8 | 10 | 1/4 | Ortho | No | Ortho | No | 5 |
| 2 | IQP | Idiopathic | L | 2 | 30 | -1 | NA | 0 | 10 | 10 | 0 | Ortho | No | ET6 | No | 3 |
| 3 | IQP | Congenital | R | 4 | 40 | -3 | NA | 4 | 8 | 10 | 1/4 | Ortho | No | Ortho | No | 1 |
| 4 | IQP | Tumor | R | 1 | 50 | -1 | -1 | 3 | 10 | 10 | 0 | Ortho | No | Ortho | No | 11 |
| 5 | IQP | Congenital | R | 68 | 60 | -2 | -2 | 5 | 10 | 10 | 0 | XT25 | No | XT25 | No | 10 |
| 6 | IQP | Congenital | R | 4 | 40 | -1 | 0 | 0 | 8 | 10 | 1/4 | Ortho | No | Ortho | No | 33 |
| 7 | IQP | Idiopathic | L | 20 | 50 | -3 | -2 | 5 | 10 | 10 | 1/2 | ET16 LHT18 | Yes | ET16 LHT12 | Yes | 5 |
| 8 | IQP | Idiopathic | R | 3 | 35 | -1 | -1 | 3 | 8 | 12 | 1/2 | ET8 | No | ET4 | No | 1 |
| 9 | LRBP | Idiopathic | R | 1 | 30 | 0 | -2 | 4 | 8 | 10 | 3 mm | Ortho | No | Ortho | No | 8 |
| 10 | LRBP | Tumor | R | 1.5 | 35 | -3 | -1 | 3 | 8 | 10 | 3 mm | XT14 | No | XT2 | No | 9 |
| 11 | LRBP | Hemorrhage | R | 4 | 70 | -7 | -3 | 6 | 10 | 10 | 3 mm | RHoT10 | Yes | RHoT10 | Yes | 2 |
| 12 | LRBP | Tumor | R | 26 | 85 | -3 | -4 | 7 | 10 | 10 | 0 mm | ET20 RHoT30 | Yes | ET20 RHoT16 | Yes | 14 |
| 13 | LRBP | Idiopathic | R | 1 | 40 | 0 | 0 | 4 | 8 | 10 | 3 mm | Ortho | No | Ortho | No | 3 |
| 14 | LRBP | Idiopathic | L | 5 | 30 | -1 | -3 | 6 | 8 | 10 | 3 mm | XT4 | No | Ortho | No | 12 |
| 15 | LRBP | Idiopathic | R | 1 | 55 | 0 | -2 | 6 | 9 | 10 | 2 mm | ET18 | No | ET25 | No | 18 |
| 16 | LRBP | Idiopathic | L | 3 | 35 | -2 | -2 | 6 | 8 | 10 | 3 mm | ET2 LHT6 | No | ET2 LHT6 | No | 5 |
| 17 | LRBP | Idiopathic | L | 2 | 45 | 0 | -1 | 5 | 8 | 10 | 3 mm | Ortho | No | Ortho | No | 4 |
| 18 | HMP | Tumor | L | 2 | 35 | -1 | -2 | 4 | 8 | 10 | 7 mm | XT2 | No | XT2 | No | 13 |
| 19 | HMP | Idiopathic | L | 14 | 40 | -2 | 0 | 4 | 8 | 10 | 7 mm | ET6 | No | ET6 | No | 1 |
| 20 | HMP | Idiopathic | R | 2 | 50 | -1 | 0 | 4 | 9 | 10 | 7 mm | Ortho | No | Ortho | No | 3 |
| 21 | HMP | Idiopathic | L | 2 | 45 | -2 | -2 | 6 | 9 | 10 | 7 mm | XT2 RHT2 | No | Ortho | No | 7 |
| 22 | HMP | Idiopathic | L | 2 | 35 | -1 | 0 | 4 | 8 | 10 | 7 mm | Ortho | No | Ortho | No | 1 |
| 23 | HMP | Idiopathic | L | 1 | 35 | -1 | 0 | 4 | 8 | 10 | 7 mm | Ortho | No | Ortho | No | 4 |
| 24 | HMP | Tumor | L | 12 | 50 | -6 | -5 | 6 | 10 | 10 | 7 mm | Ortho | No | Ortho | No | 1 |
| 25 | HMP | Idiopathic | R | 3 | 50 | -1 | 0 | 4 | 9 | 10 | 8 mm | Ortho | No | ET16 | No | 4 |
| 26 | HMP | Idiopathic | L | 20 | 75 | -2 | -3 | 7 | 10 | 10 | 7 mm | ET25 | No | ET25 | No | 1 |
| 27 | HMP | Tumor | L | 1 | 50 | -6 | -2 | 6 | 10 | 10 | 7 mm | XT2 | No | XT2 | No | 1 |

ABD, abduction; CD, circumferential displacement of the anchoring site; ET, esotropia; F, female; FDT, forced duction test; F?U, follow-up; HMP, horizontal meridian positioning; HoT, hypotropia; HT, hypertropia; IQP, intra-quadrant positioning; L, left; LRBP, lateral rectus border positioning; M, male; ML, length of the muscle to be transposed; N/A, not available; PD, prism diopters; R, right; RD, radial distance from limbus to the anchoring site; SD, standard deviation; XT exotropia
